# Supplementary material for: Empirical methods for controlling false positives and estimating confidence in ChIP-Seq peaks
Source: BMC Bioinformatics. 2008 Dec 5;9:523. doi: 10.1186/1471-2105-9-523 (PMC2628906; doi:10.1186/1471-2105-9-523)
Supplement: Additional File 1 — A variety of html documents from the USeq web site detailing the available applications, their best usage, output file type descriptions, command line menus, etc. [file 1471-2105-9-523-S1.zip › USeqUserGuides/JavaDoc.zip!/JavaDoc/util/bio/parsers/MultiFastaParser.html]

MultiFastaParser


|  |  |  |
| --- | --- | --- |
| **Overview** \| **Package** \| **Class** \| **Use** \| **Tree** \| **Deprecated** \| **Index** \| **Help** | |  |
| **PREV CLASS**   **NEXT CLASS** | **FRAMES**    **NO FRAMES**     **All Classes** |  |
| SUMMARY: NESTED \| FIELD \| CONSTR \| METHOD | DETAIL: FIELD \| CONSTR \| METHOD |  |


---


## util.bio.parsers Class MultiFastaParser

```
java.lang.Object
  util.bio.parsers.MultiFastaParser
```

---

``` public class MultiFastaParser extends java.lang.Object ```

Extracts sequences and headers out of multi fasta files.
Will read in combine fasta files, skips blank lines, does not filter actual seq
parses off the > character. Seqs and names are index matched, ie seqs[3] and names[3] refer
to the same thing.

---

**Constructor Summary**

`MultiFastaParser()`

`MultiFastaParser(java.io.File file)`

`MultiFastaParser(java.io.File[] fastaFiles)`

`MultiFastaParser(java.io.File file,
int minLength,
boolean justPrint)`

`MultiFastaParser(java.lang.String file)`


| **Method Summary** | |
| --- | --- |
| `Fasta[]` | `getFastas()` |
| `int` | `getMinLength()` |
| `java.lang.String[]` | `getNames()` |
| `java.util.ArrayList` | `getNamesAL()` |
| `java.util.HashMap` | `getNamesSeqs()`             Returns a hashmap of the fasta name: sequence |
| `int` | `getNumReads()` |
| `java.lang.String[]` | `getSeqs()` |
| `java.util.ArrayList` | `getSeqsAL()` |
| `java.util.HashMap` | `getSeqsNames()`             Returns a hashmap of the fasta sequence:name |
| `boolean` | `isFastaFound()` |
| `boolean` | `isJustPrint()` |
| `void` | `parseIt(java.io.File file)`             Every call adds to the list of sequences and names. |
| `void` | `printFasta()` |
| `void` | `resetFields()` |
| `void` | `setFastaFound(boolean fastaFound)` |
| `void` | `setJustPrint(boolean justPrint)` |
| `void` | `setMinLength(int minLength)` |
| `void` | `setNamesAL(java.util.ArrayList namesAL)` |
| `void` | `setSeqsAL(java.util.ArrayList seqsAL)` |

**Methods inherited from class java.lang.Object**

`equals, getClass, hashCode, notify, notifyAll, toString, wait, wait, wait`

**Constructor Detail**

### MultiFastaParser

```
public MultiFastaParser(java.io.File file)
```

---


### MultiFastaParser

```
public MultiFastaParser(java.io.File file,
                        int minLength,
                        boolean justPrint)
```

---


### MultiFastaParser

```
public MultiFastaParser(java.lang.String file)
```

---


### MultiFastaParser

```
public MultiFastaParser()
```

---


### MultiFastaParser

```
public MultiFastaParser(java.io.File[] fastaFiles)
```


**Method Detail**

### resetFields

```
public void resetFields()
```

---


### parseIt

```
public void parseIt(java.io.File file)
```

:   Every call adds to the list of sequences and names.

---


### printFasta

```
public void printFasta()
```

---


### getNamesSeqs

```
public java.util.HashMap getNamesSeqs()
```

:   Returns a hashmap of the fasta name: sequence

---


### getSeqsNames

```
public java.util.HashMap getSeqsNames()
```

:   Returns a hashmap of the fasta sequence:name

---


### getSeqs

```
public java.lang.String[] getSeqs()
```

---


### getNames

```
public java.lang.String[] getNames()
```

---


### getFastas

```
public Fasta[] getFastas()
```

---


### getNumReads

```
public int getNumReads()
```

---


### isFastaFound

```
public boolean isFastaFound()
```

---


### getMinLength

```
public int getMinLength()
```

---


### setMinLength

```
public void setMinLength(int minLength)
```

---


### isJustPrint

```
public boolean isJustPrint()
```

---


### setJustPrint

```
public void setJustPrint(boolean justPrint)
```

---


### getNamesAL

```
public java.util.ArrayList getNamesAL()
```

---


### setNamesAL

```
public void setNamesAL(java.util.ArrayList namesAL)
```

---


### getSeqsAL

```
public java.util.ArrayList getSeqsAL()
```

---


### setSeqsAL

```
public void setSeqsAL(java.util.ArrayList seqsAL)
```

---


### setFastaFound

```
public void setFastaFound(boolean fastaFound)
```


---


|  |  |  |
| --- | --- | --- |
| **Overview** \| **Package** \| **Class** \| **Use** \| **Tree** \| **Deprecated** \| **Index** \| **Help** | |  |
| **PREV CLASS**   **NEXT CLASS** | **FRAMES**    **NO FRAMES**     **All Classes** |  |
| SUMMARY: NESTED \| FIELD \| CONSTR \| METHOD | DETAIL: FIELD \| CONSTR \| METHOD |  |


---
